# *Adansonia digitata* L. (Baobab) Bioactive Compounds, Biological Activities, and the Potential Effect on Glycemia: A Narrative Review

**DOI:** 10.3390/nu15092170

**Published:** 2023-05-01

**Authors:** Maria Leonor Silva, Keyla Rita, Maria Alexandra Bernardo, Maria Fernanda de Mesquita, Ana Maria Pintão, Margarida Moncada

**Affiliations:** Centro de Investigação Interdisciplinar Egas Moniz, Instituto Universitário Egas Moniz, Campus Universitário, Quinta da Granja, Monte de Caparica, 2829-511 Caparica, Portugal; keyla.rita@gmail.com (K.R.); abernardo@egasmoniz.edu.pt (M.A.B.); fmesquita@egasmoniz.edu.pt (M.F.d.M.); apintao@egasmoniz.edu.pt (A.M.P.); margaridacm@egasmoniz.edu.pt (M.M.)

**Keywords:** *Adansonia digitata* L., baobab, traditional uses, polyphenols, tannins, fiber, biological activities, glycemia, diabetes mellitus

## Abstract

*Adansonia digitata* L. fruit, also known as baobab, has been used traditionally throughout the world for its medicinal properties. Ethnopharmacological uses of various plant parts have been reported for hydration, antipyretic, antiparasitic, antitussive, and sudorific properties and also in the treatment of diarrhea and dysentery in many African countries. Several studies have revealed that in addition to these applications, baobab has antioxidant, anti-inflammatory, analgesic, and antimicrobial activities. The health benefits of baobab have been attributed to its bioactive compounds, namely phenols, flavonoids, proanthocyanins, tannins, catechins, and carotenoids. Baobab fruit is also an important source of vitamin C and micronutrients, including zinc, potassium, magnesium, iron, calcium, and protein, which may reduce nutritional deficiencies. Despite scientific studies revealing that this fruit has a wide diversity of bioactive compounds with beneficial effects on health, there is a gap in the review of information about their mechanisms of action and critical analysis of clinical trials exploring, in particular, their effect on glycemia regulation. This work aims to present a current overview of the bioactive compounds, biological activities, and effects of *A. digitata* fruit on blood glucose, highlighting their potential mechanisms of action and effects on glycemia regulation, evaluated in recent animal and human trials.

## 1. Introduction

Aging is one of the most important demographic problems worldwide [1]. One in six people will be 60 years or older worldwide by 2030, and two-thirds of the world’s population will be over 60 by 2025 [2]. Furthermore, multimorbidity is a global phenomenon associated with aging, which includes one or more of six conditions, namely, arthritis, heart disease, asthma, depression, schizophrenia or psychosis, and diabetes [3]. Diabetes is a metabolic disease that has a silent and adverse impact on the health of the elderly [4]. In this sense, it is important to implement effective strategies for blood glucose control in older people [5].

The *Adansonia digitata* L. tree is considered emblematic and essential in traditional medicine in Africa and India [6]. Its existence is believed to have been known for over 4000 years [7], first described in Senegal in the 18th century [6]. The genus *Adansonia* belongs to the *Malvaceae* family, and eight species have been identified in many African countries [6]. The species *Adansonia digitata* L. is native to the African continent [8], being found mainly in the savannas of sub-Saharan Africa at low altitudes, with 4 to 10 months of drought per year [9]. Both the tree and the fruit are universally known as baobab [6]; however, the tree is also known as “magic tree,” “chemist tree,” or “symbol of the earth” [6]. This fruit plays an important role as a livelihood for rural communities [9]. Food products processed from baobab have been used for their cultural importance and include pasta, porridge, beverages, sauces, flavoring agents, and others [9]. Baobab use has increased worldwide in the medical, food, and cosmetic industries [9].

**Figure 1:**
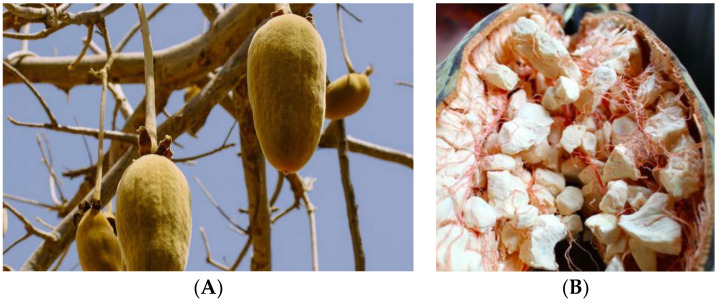
Whole fruit of *Adansonia digitata* L. (**A**) and epicarp, mesocarp, and endocarp with seeds and red filaments (**B**).

Baobab fruits are large, ovoid [8], and irregular in shape, consisting of the pericarp and seeds [7]. Externally, the epicarp (peel), corresponding to 45% wet weight of the fruit [10], is a wide, hard, velvety, and brownish-green casing [6] (Figure 1). Internally, there is a white, floury pulp with an acrid flavor (mesocarp and endocarp), constituting 15% wet weight of the fruit [10], intercepted by red filaments involving many seeds clustered together [11] that are equivalent to 40% wet weight of the fruit [10]. This fruit is usually ingested as fresh fruit or as a food supplement in capsules, pulverized pulp, or drinks [10]. For medicinal purposes, all fruit except the epicarp is boiled, filtered, and its aqueous extract drunk [10].

*A. digitata* L. fruit, seeds, bark, and leaves have been traditionally used by African populations due to their nutritional characteristics and pharmacological activities [6]. According to Braca (2018), a variability of baobab from different origins revealed a promising source of components with benefits [12]. A number of phytochemical compounds have been identified in distinct parts of *Adansonia digitata* L., which have been demonstrated to possess beneficial effects in different diseases. This plant has been shown to exert hypoglycemic, hypolipidemic, antimicrobial, analgesic, and antipyretic activities [6,7,11,12,13,14,15]. Furthermore, it is a hepatoprotective agent with anti-inflammatory and antioxidant properties [6,7,11,13,14,15]. 

Polyphenols are phytochemical compounds that have been shown to be effective as diabetic agents through the regulation of glucose homeostasis [16]. Different mechanisms of action have been described, namely, insulin secretion improvement and glucagon-like-peptide (GLP-1) secretion, which stimulates postprandial insulin secretion [16]. The potential effect of this fruit in glycemia management has been attributed to procyanidin compounds that activate insulin signaling pathways and glucose transport to the adipose and muscle cells [17]. Results from animal and human studies have shown *Adansonia digitata* L. fruit aqueous extract ingestion to be effective in blood glucose level control [18,19]. 

Hyperglycemia is one of the major risk factors for type 2 diabetes mellitus and cardiovascular disease development [20]. According to Luc et al. (2019), high blood glucose levels can produce reactive oxygen species, which promote an oxidative stress status and vascular dysfunction [21]. The molecular mechanism underlying hyperglycemia consequences can be related to the upregulation of proinflammatory and oxidative stress markers, which can lead to impaired insulin secretion and insulin resistance [21].

The present review provides an overview of *Adansonia digitata* L bioactive compounds, biological activities, and their potential effect on glycemia. 

## 2. Nutritional Composition

The nutritional composition of *A. digitata* L. fruit (Table 1) shows that this fruit is rich in fiber (80.3/100 g dry fruit) [22], which could beneficially contribute to blood glucose management [23]. According to Reynolds et al. (2020), diets with high fiber content demonstrate improved glycemic control. These authors suggest a daily fiber intake of around 35 g to reduce the risk of mortality in diabetic adults [23]. The fruit of *A. digitata* L. contains a high amount of both soluble and insoluble dietary fiber. The pulp contains 16.1 (±0.8) g/100 g of insoluble fiber and 57.3 (±0.3) g/100 g of soluble fiber, and the seeds contain 62.0 (±1.2) g/100 g of insoluble fiber and 16.3 (±0.6) g/100 g of soluble fiber [22].

Baobab also has a relevant content of micronutrients, such as a high content of potassium (1240 mg/100 g fresh fruit pulp) [24] and phosphorus (775 mg/100 g dry fruit pulp) [25] (Table 1). Plasma potassium and phosphorus levels have been inversely associated with risk factor development for type 2 diabetes mellitus due to their important role as mediators in glucose metabolism [26,27]. 

The ingestion of baobab can also beneficially contribute to health due to iron (14.97 mg/100 g) and zinc (1.8 mg/100 g) content. These micronutrients have been associated with decreased risk of cardiovascular disease and type 2 diabetes mellitus [28]. Evidence from human studies have shown that an imbalance of iron leads to impaired glucose metabolism [29]. Moreover, baobab is rich in other micronutrients, such as sodium, magnesium, potassium, and calcium (Table 1). According to Dubey, 2020, these micronutrient deficiencies can be directly or indirectly associated with insulin resistance or type 2 diabetes through oxidative stress [29]. 

Baobab also has a high content of vitamin C (466 mg/100 g), Table 1. However, according to Monteiro et al. (2022), baobab nutritional composition, especially protein, sugar, and vitamin C content, can significantly differ according to different samples obtained in different locales [30].

**Table 1:** Nutritional composition of *Adansonia digitata* L. fruit.

Nutritional Composition	g/100 g Dry ^1^ or Fresh ^2^ Weight of L. Fruit Pulp	Reference
Starch ^1^	39.2	[8]
Glucose ^1^	7.9	[8]
Fructose ^1^	7.0	[8]
Sucrose ^1^	1.7	[8]
Protein ^1^	3.0	[8]
Lipid ^1^	0.5	[8]
Fiber ^1^	80.3	[22]
	mg/100 g Dry ^1^ or Fresh ^2^ Weight of L. Fruit Pulp	
Calcium ^1^	309 ± 1	[25]
Iron ^1^	14.97 ± 0.06	[25]
Sodium ^1^	34.61 ± 0.3	[25]
Magnesium ^1^	155 ± 2	[25]
Phosphorus ^1^	775 ± 2	[25]
Zinc ^2^	1.8 ± 0	[25]
Potassium ^2^	1240 ± 40	[24]
Vitamin C ^1^	466 ± 2.55	[31]

^1^ mg/100 g dry; ^2^ mg/100 g Fresh

## 3. Phytochemical Compounds

Phytochemical compounds present in foods have long been related to beneficial properties in human health, namely, their hypoglycemic and antidiabetic effects [32]. *Adansonia digitata* L. fruit is rich in several bioactive compounds, as reported in several studies and shown in Table 2. 

**Figure 2:**
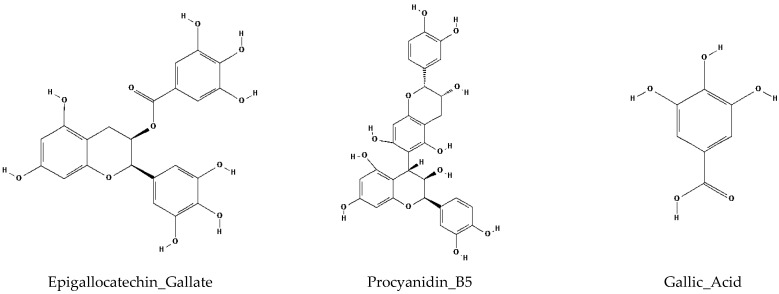

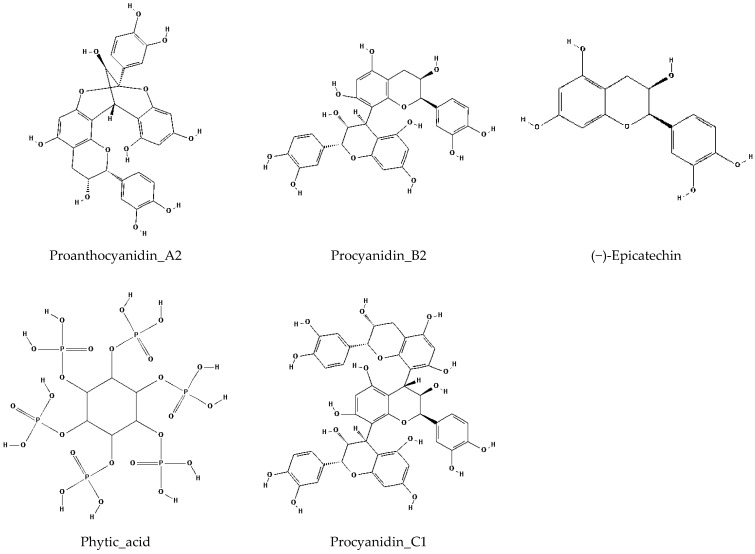
Chemical structures of the main compounds identified in *Adansonia digitata* L. fruit.

The main compounds found in baobab fruit are phenols, namely, gallic acid and epicatechin-(4β→8)-epicatechin (procyanidin B2), Figure 2 [33]. Other polyphenols have been identified in the baobab fruit [33], namely, the procyanidins (−)-epicatechin, epicatechin-(2β→O→7, 4β→8)-epicatechin (procyanidin A2), epicatechin-(4β→6)-epicatechin (procyanidin B5), epicatechin-(4β→8)-epicatechin (4β→8)-epicatechin (procyanidin C1) [35], and epigallocatechin-3-gallate (EGCG) (Figure 2) [33] and flavonoids such as quercetin [36]. Procyanidins are polyphenols widely distributed in plants that possess anti-inflammatory and antioxidative effects related to the pathophysiology of insulin resistance and type 2 diabetes mellitus [37]. Clinical trials suggest that polyphenols naturally present in the diet improve glucose metabolism [38]. Catechins contribute to reducing glycemia, enhancing insulin sensitivity, decreasing blood lipids, and reducing white fat depot [37]. Gallic acid is a phenolic acid that has been shown to possess antioxidant and antiglycation properties, which could act beneficially in diabetes [39]. 

In *Adansonia digitata* L. pulp have also been identified high concentrations of vitamin C [34] and organic acids [22], triterpenes, steroids, saponins [13], 5-hydroxymethylfurfural (5-HMF), caffeine [33,34], and carotenes [34]. In addition, *A. digitata* also contains mucilage and pectin [10]. 

The baobab fruit contains some antinutrients such as tannins, phytic acid, and trypsin inhibitors such as BAPA (*N*-benzoyl-*DL*-arginine-*P*-nitroanilide) and trypsin type III [24]. Some toxic compounds such as oxalates and hydrocyanic acid were also found [40]. 

The quantitative and qualitative phytochemical composition of *Adansonia digitata* L. fruit depends on multifactorial variations such as the plant variety and age of the tree, genetic variation, environmental factors, namely, the type of soil and its chemical composition, fertilization, exposure to sunlight, water supply, sample collection, storage method, and subsequent chemical analysis methods [40].

## 4. Ethnobotanical Uses and Pharmacological Applications

The baobab tree has great utility and importance for the livelihood of rural people. The fruit pulp (mesocarp and endocarp), filaments and seeds, extract, or powder is typically consumed by the population [41]. Usually, the fruit (except the epicarp) or the powder pulp alone is dissolved in water or milk in the preparation of sauces, infant porridge, and hydrating drinks [41].

The baobab fruit is used for medicinal purposes in the control and treatment of diseases due to its therapeutic activities [6,7,14]. Some of these therapeutic properties have been recognized by the rural population of some African countries (Table 3) and have been scientifically evaluated (Table 4). 

The pulp and seeds of *A. digitata* are used as diarrhea, dysentery, antipyretic, antiparasitic, and antitussive agents [6]. Additionally, in the Ivory Coast and eastern Africa, the pulp and seeds are used for oral hydration [6,10]. The people of Tanzania have also used the fruit decoction for hemoptysis [6].

The evidence found in both in vitro and in vivo studies (Table 4) shows that several compounds present in the pulp or in the seeds have multiple beneficial activities. Polyphenols in general and specifically tannins and flavonoids improve insulin and glycemia responses [44]. Polyphenols with ascorbic acid act in the protection against oxidative stress [11]. Mucilage and fiber contribute as oral hydration for diarrhea [42].

Triterpenes, saponins, and sterols have complementary anti-inflammatory and analgesic activities that can be found in aqueous extracts, explaining the ethnobotanical uses by the population [13].

## 5. Mechanisms of Action of *Adansonia digitata* L. Bioactive Compounds

In vitro and in vivo studies have provided information on the potential mechanisms of action of dietary polyphenols in glucose homeostasis [47]. Generally, these compounds have been shown to regulate the glycemic response through different proposed mechanisms, namely, regulating digestive enzyme activity, decreasing glucose absorption in the intestine, increasing glucose uptake in cells, and improving pancreatic beta-cell function [47]. The effects and the mechanisms of action of *A. digitata* bioactive compounds on glycemia homeostasis are synthesized in Table 5.

The bioactive components of baobab, such as flavonoids, phenolic acids, and tannins, inhibit the enzymes α-amylase and α-glucosidase, found in the small intestine, which are responsible for carbohydrate digestion [48]. These enzymes catalyze the hydrolysis of complex carbohydrates (starch, glycogen, etc.) into monosaccharides for intestinal absorption [49]. Several experiments with plant-polyphenol-rich extracts demonstrated the inhibition of digestive enzymes that can decrease glucose absorption, promoting a hypoglycemic effect [50].

Procyanidins, specifically (−)epicatechin and cyanidin-3-O-β-glucoside, have been shown to possess an anti-hyperglycemic effect through translocation of glucose transporter 4 (GLUT 4). These compounds, in concentrations of 10 µg/Kg mice body weight, activated both insulin-signaling pathways and AMPK-signaling pathways to induce GLUT4 translocation in muscle cells [17]. Epicatechin can also regulate glucose homeostasis by improvement of gene expression of glucose transporter 2 (GLUT2) in pancreatic and duodenal cells [51]. 

*Adansonia digitata* L. fruit can beneficially contribute to diabetes, not only through its glycemic control activity but also due to its antioxidant properties. Epicatechin (A2) inhibits cells’ prooxidative enzymes, such as lipoxygenase and xanthine oxidase [52]. This baobab component possesses a high capacity of free radical scavenging [52]. 

According to Yin et al. (data), procyanidin B2 seems to be effective as an anti-inflammatory agent of pancreatic cells of diabetic animals, and the administration of this component can exhibit a protective effect by interleukin-1 regulation [53]. Epigallocatechin-3-gallate (EGCG) increases glucose transporter translocation in L6 cells [54].

Other baobab bioactive compounds such as gallic acid can also act beneficially on diabetes through glucose and insulin homeostasis improvement [55]. Plant-polyphenol-rich extracts have increased glucagon-like peptide (GLP-1) concentration after a nutrient load in an animal model. Although this mechanism is not completely understood, polyphenols seem to exert a stimulation of incretins, which can contribute to the management of blood glucose and insulin levels [16]. Phytic acid decreased blood glucose levels and oxidative stress by scavenging free radical activity [56].

**Table 5:** Summary of the effects and mechanisms of action of the bioactive compounds of *Adansonia digitata* L.

Bioactive Compound	Effect	Mechanism of Action	Reference
(–)-epicatechin	Increase glucose transporter into cells Decrease blood glucose level Decrease oxidative stress	↑ GLUT4 ↑ PI3K/Akt ↑ AMPK ↓ ERO	[17,57]
Epicatechin-(2β→O→7, 4β→8) -epicatechin (A2)	Decrease blood glucose level Decrease pancreatic apoptosis Increase insulin secretion Increase glucose homeostasis	↑GLUT2 mRNA ↑ Pdx1	[51]
Epicatechin-(4β→8)-epicatechin (B2)	Increase insulin level Increase islet sizes Decrease inflammation Decrease oxidative stress	↓ Alpha-glucosidase activity ↓ AGE ↓ ROS ↓ IL-1β ↓ 15-LO ↓ XO	[17,52,53]
Epicatechin-(4β→6)-epicatechin (B5)	↓ Blood glucose level	↓ alpha-glucosidase	[52]
Epicatechin-(4β→8)-epicatechin-(4β→8)-epicatechin (C1)	Increase glucose transporter into cell ↓ Blood glucose level ↓ Oxidative stress	↑ GLUT4 ↑ PI3K/Akt ↓ α-glucosidase ↑ AMPK ↓ ROS ↓ 15-LO	[17,52]
Epigallocatechin-3-gallate (EGCG)	Increase glucose transporter ↓ Insulin resistance	↑ GLUT4 ↑AMPK ↑ PI3K/Akt	[54]
Gallic acid (3, 4, 5-trihydroxybenzoic acid)	Improve insulin homeostasis Improve glucose homeostasis	↑ AMPK ↑ PGC1alpha	[55]
Phytic acid	↓ Blood glucose level ↓ Oxidative stress	↓ α-glucosidase ↓ α-amylase ↓ Scavenging free radicals	[56]

Pdx1—pancreatic and duodenal homebox; GLUT2—glucose transporter 2; GLUT4—glucose transporter 2; PI3K/Akt—phosphatidylinositol 3-kinase; AMPK—AMP-activated protein kinase; PGC1—peroxisome proliferator-activated receptor coactivated 1; ROS—reactive oxygen species; IL-1—interleukin-1; LO—lipoxygenase; XO—xanthine oxidase; AGE—advanced glycation end-product; ↓—increase; ↓—decrease

## 6. Antidiabetic Activities of *Adansonia digitata* L.

### 6.1. In Vitro Studies

Coe et al. (2013) showed that baobab extracts significantly reduced starch breakdown in vitro, resulting in a decreased sugar release from white bread samples, at 20 and 60 min, compared to control samples [58]. Baobab is rich in polyphenol compounds, which inhibited the digestive enzyme activity in vitro in different concentrations [59]. According to Vosloo et al. (2005), polyphenol extract is particularly potent, with an IC50 value of 4.5 µg GAE (gallic acid equivalent)/mL in starch digestion modulation, through inhibition of alpha-amylase and alpha-glucosidase activities. Additionally, baobab possesses a high content of fiber that may also contribute to decreased sugar release [60]. Saravanaraj et al. (2017) also verified that *A. digitata* L. fruit administration increased the number and size of the islets of Langerhans, the presence of normal pancreatic cells, and the regeneration of necrosis and fibrosis caused by diabetes [61].

### 6.2. In Vivo Studies

The potential use of *Adansonia digitata* L. in glycemia control has been investigated in different animal models, suggesting that it possesses an antidiabetic effect in vivo [19,36], Table 6. The traditional use of baobab pulp has sparked the scientific interest of many researchers in determining its pharmacological activities [61]. *A. digitata* has been shown to possess hypoglycemic properties, but, to date, few scientific studies have been conducted that reveal this activity [18,58].

The antidiabetic activity of the aqueous fruit pulp extract of *Adansonia digitata* L. was investigated in alloxan-induced diabetic rats by Muhammad and co-workers, who found that after 2 weeks of administration (10, 100, and 1000 mg/kg), blood glucose was significantly lower (*p* < 0.05) in the intervention group compared to the control group [62] (Table 6). Furthermore, Gwarzo and Bako (2013) observed a significant reduction in serum glucose in alloxan-induced diabetic rats after methanolic extract of *A. digitata* fruit pulp administration (100, 200, and 300 mg/Kg) once daily for 4 weeks. At days 14 and 28, there was a significant reduction in serum glucose (*p* < 0.001) in the intervention groups (with all doses) compared to the control group [36]. This study demonstrates that this fruit has a potential hypoglycemic effect not only in acute conditions but also with long-term administration (Table 6). 

Tanko and collaborators (2008) reported that the methanolic stem bark extract of *A. digitata* significantly decreased (*p* < 0.05) blood glucose levels in streptozotocin-induced diabetic Wistar rats compared to the control group. Specifically, the ingestion of 100, 200, and 400 mg/kg significantly improved plasma glucose levels (*p* < 0.05) after 3, 5, and 7 h [19] (Table 6). 

The hypolipidemic effect of *A. digitata* leaves methanolic extract (200 and 400 mg/Kg) was also evaluated in diabetic rats for 6 weeks [63]. Oral treatment with this extract initiated a significant reduction in fasting blood glucose level and glycosylated hemoglobin (*p* < 0.05), confirming that the leaves’ active compounds can act beneficially in diabetes management. According to Wang et al. (2021), hemoglobin A1c is a factor that contributes to diabetes complication development [65]. Pamela and co-authors (2019) also demonstrated that the ingestion of aqueous leaf extract (200 mg/kg, 400 mg/Kg, 600 mg/kg) for 2 weeks significantly decreased fasting blood glucose level in alloxan-induced diabetic rats [64].

The ingestion of *A. digitata* fruit also contributed to improved (*p* < 0.05) lipid profiles, including cholesterol, triglycerides, high-density lipoprotein, and low-density lipoprotein, compared to the diabetic control group [63] (Table 6). As diabetes can lead to disease-related complications such as cholesterol metabolism dysfunction [66], ingestion of *A. digitata* extracts could beneficially act in diabetes complications.

### 6.3. Human Clinical Trials

There are few clinical trials described in the literature, but they suggest a benefit in blood glucose levels (Table 6). Coe et al. (2013) verified that the consumption of baobab fruit aqueous extract from five different locations in Africa in two doses (18.5 g and 37 g) did not significantly reduce blood glucose response when compared to the control group [58]. However the incremental area under the curve (AUCi) of glucose response significantly decreased, at each point, with baobab extract intake compared to the control group in nondiabetic subjects. Similarly, Keyla et al. (2022) found that aqueous Angolan baobab fruit extract significantly lowered AUCi (0–120 min) in the intervention group (0.13 g/mL baobab extract fresh weight) compared to the control group (*p* = 0.012) in healthy subjects [18] (Table 6).

According to Coe et al. (2013), despite the promising effect found on glycemia, the fruit extract did not reveal an effect on energy expenditure and satiety in nondiabetic adults [58]. Different factors could contribute to these results, since satiety is dependent on diverse aspects such as weight, volume, energy and nutrient content of meals, and energy density of meals, among others [67]. In this context, further studies with a larger sample and well-controlled clinical trials should be employed to evaluate the effects of baobab extract on satiety and energy expenditure. 

For total insulin response in healthy participants, the extract of the baobab fruit originates a significantly lower (*p* < 0.05) area under the curve [44]. Table 6 summarizes the effect of *A. digitata* results on glucose metabolism and other parameters of animal and human models.

Data from the literature should be analyzed carefully, since heterogeneity regarding food intake parameters could represent a bias [44,58]. Glycemia and insulinemic metabolism depends on various factors such as meal composition, timing, and quantity of food ingested [68,69]. In this context, more studies need to be designed with a well-controlled food daily intake in order to evaluate the effect of this fruit on glucose metabolism.

The protein and lipid contents can delay gastric emptying and stimulate insulin production, exerting regulatory action on postprandial blood glucose [22,70]. Meal fiber content can also significantly influence glycemia [68]. High fiber content shows a beneficial effect on satiety by providing slow emptying of gastric contents [71]. According to Garvey et al. (2017), administration of 15 g of baobab pulp significantly increased satiety in healthy adults [70]. The authors suggest that this effect may be due to delayed gastric emptying caused by the fiber and polyphenol contents in the fruit [70]. 

## 7. Conclusions

This review provides evidence that *Adansonia digitata* L. fruit is an important source of gallic acid, (-)-epicatechin, epicatechin-3-gallate, procyanidin B2, tannins and carotenoids. These bioactive compounds seem to exert beneficial effects on health due to antioxidant and anti-inflammatory properties. This work highlights the beneficial effect on diabetes through different mechanisms of action, namely, decreasing digestive enzyme activities, translocation of glucose transporter 4 into cells, and activation of insulin and the AMPK-signaling pathway. Clinical study results indicate promising applications in the treatment of diabetes with *Adansonia digitata* L. aqueous extracts. However, there is a lack of randomized clinical trials on biochemical parameters in diabetic or impaired glycemia subjects. Further studies should be conducted to verify the effect of this fruit as part of a daily diet and for a longer period. However, studies have shown the excellent nutritional profile of this fruit, which suggests that its intake can help reduce nutritional deficiencies.

## Figures and Tables

**Table 2:** Main phytochemical compounds of *Adansonia digitata* L. fruit.

Compounds	mg/100 g Fresh Weight of L. Fruit Pulp	Reference
Gallic acid	68.54 ± 12.4	[33]
(–)-epicatechin	43 ± 3.08	[33]
Epicatechin-3-Gallate	9.98 ± 0.08	[33]
Procyanidin B2	533.30 ± 22.6	[33]
Condensed tannins	336.33 ± 10.85	[18]
Hydrolysable tannins	237.63 ± 4.71	[18]
Total phenols	702.39 ± 11.85	[18]
Total carotenoids	0.29 ± 0.5	[34]
Caffeine	0.87 ± 0.04	[34]
Phytic acid	2.6 ± 0.4	[24]
Trypsin inhibitors	5.9 ± 0.3	[24]

**Table 3:** Ethnopharmacological uses of *Adansonia digitata* L. fruit.

Part Used	Traditional Use	Country	Reference
Pulp aqueous extract	Oral hydration for diarrhea and dysentery	India	[42]
Seeds with water	Oral hydration for diarrhea and dysentery	South Africa	[6]
	Antipyretic agent	South Africa	[6]
Seeds and fruit decoction	Oral hydration for diarrhea and dysentery	Tanzania, Cameroon, Central Africa	[6]
	Hemoptysis	Tanzania	[6]
	Antipyretic agent	Tanzania, Cameroon, Central Africa	[6]
Pulp and seeds	Oral hydration for diarrhea and dysentery	Ivory Coast	[6]
	Antiparasitic agent against worms	Ivory Coast	[6]
Pulp and seeds aqueous extract	Neutralization of *Strophanthus* genus toxic compounds	Eastern Africa	[10]
Seed oil	Hydration in cases of eczema and psoriasis	Africa	[43]
Seed powder	Antitussive agent	South Africa	[6]
Seeds	Oral hydration	Eastern Africa	[10]
Pulp and seed powder	Sudorific agent	Eastern Africa	[10]
Leaves	Toothache, gingivitis	Burkina Faso	[6]
Leaves	Diaphoretic, fever remedy	Kenya	[6]
Fruit, seeds	Diuretic, refreshing	Tanzania	[6]
Leaves	Malaria	Sierra Leone	[6]

**Table 4:** Studies evaluating the effect of *Adansonia digitata* L. fruit phytochemicals compounds.

Part Used	Effect	Bioactive Compounds	Reference
Pulp aqueous extract	Analgesic and anti-inflammatory	Sterols, saponins, and triterpenes	[13]
	Antipyretic	Sterols, saponins, e triterpenes	[13]
	Oral hydration for diarrhea	Tannins, mucilage, cellulose, and citric acid	[42]
	Anti-inflammatory	Tannins, mucilage, cellulose, and citric acid	[42]
	Improve insulin response	Tannins and flavonoids	[44]
	Improve glycemia response	Polyphenols and soluble fiber	[44]
	Anti-inflammatory, analgesic, immunostimulant Antimicrobial agent	Triterpenes, β–sitosterol, palmitate β–amirine, and ursolique acid	[14]
	Stimulation of growth and metabolic activity of beneficial bacteria	Soluble fiber	[42,45]
Pulp aqueous extract and red fibers	Protection against oxidative stress and immunity increase in chronic illness	Polyphenols and ascorbic acid	[11]
Extracts of fruit powder and fibers	Protection against proliferation of human colon cancer	-	[46]
Seed oil	Regenerator, moisturizing, and smoothing of the skin	Vitamins, fat acids, and sterols	[6,31]
	Regeneration agent on epithelial tissue; promotion of skin tone and elasticity; analgesia	Vitamins	[6]
	Oral and dental cleaning tool	-	[5]
Pulp and seed extract	Antibacterial agent against *Bacillus subtilis*, *Escherichia coli*, and *Mycobacterium leprae*	-	[45]
Seed aqueous extract	Anti-inflammatory in rheumatic diseases	-	[7]

**Table 6:** Effects of *Adansonia digitata* L. on glucose metabolism in animal and human models.

References	Study Design	Samples	Interventions	Outcomes
[19]	Clinical controlled trial	Streptozotocin-induced diabetic rats (*n* = 25)	Rhytidome methanolic extract (100, 200, and 400 mg/kg), 7 h	↓ Blood glucose level (*p* < 0.05)
[36]	Clinical controlled trial	Alloxan-induced diabetic rats (*n* = 40)	Pulp methanolic extract (100, 200, and 300 mg/kg per day), 28 days	↓ Blood glucose level (*p* < 0.05)
[62]	Clinical controlled trial	Alloxan-induced diabetic rats (*n* = 36)	Pulp aqueous extract (1, 2, and 3 g/kg per day), 14 days	↓ Blood glucose level (*p* < 0.05)
[61]	Clinical controlled trial	Diabetic rats (*n* = 30)	Pulp ethanolic extract (200 and 400 mg/kg per day) 28 days	Necrosis and fibrosis improvement; Increase of pancreatic beta-cell number and size
[63]	Clinical controlled trial	Streptozotocin-induced diabetic rats (*n* = 40)	Leaf methanolic extract (5, 200, 400 mg/kg), 6 weeks	Improved body weight (*p* < 0.05) ↓ Total cholesterol level (*p* < 0.05)
[64]	Clinical controlled trial	Alloxan-induced diabetic rats (*n* = 36)	Aqueous leaf extract (200, 400, 600 mg/kg), 2 weeks	↓ Fasting blood glucose level (*p* < 0.05)
[58]	Randomized, blind, controlled trial	Healthy female subjects (*n* = 9)	Pulp aqueous extract (18.5 g/in 250 mL water; 37 g/in 250 mL water), 3 days	↓ Postprandial blood glucose with both doses (*p* < 0.05) No effect on satiety
[44]	Randomized, blind, controlled trial	Overweight male and female subjects (*n* = 13)	Pulp with bread (1.8% in 106.97 g of bread) 1 time with bread	↓ Total insulin response—area under the curve (*p* < 0.05)
[18]	Randomized, controlled trial	Impaired fasting glycemia subjects (*n* = 22)	Aqueous extract (0.13 g/mL extract fresh weight), 1 time (OGTT)	↓ glycemia incremental area under the curve (*p* = 0.012) ↓ glucose maximum concentration (*p* = 0.029)

OGTT—oral glucose tolerance test; ↑—increase; ↓—decrease.

## Data Availability

Not applicable.
